# Fluorescent carbon dots in PEC-GS/BG hybrids and their application for bioimaging

**DOI:** 10.3389/fmolb.2025.1555995

**Published:** 2025-06-19

**Authors:** Xibing Zhang, Jun Gong, Hai Zhou, Xun Yin, Guanda Wu, Qixiang Wang, Weipeng Lin, Huaguo Wang, Wei Ji, Zhongmin Zhang

**Affiliations:** Department of Spine and Orthopedics, The Third Affiliated Hospital of Guangzhou University of Chinese Medicine, Guangzhou, China

**Keywords:** bioimaging, bioactive glass, carbon dots, citric acid, photoluminescence

## Abstract

Carbon dots (CDs), renowned for their distinctive photoluminescence properties, have emerged as a prominent material in the field of luminescence. They are extensively utilized in bioimaging, drug delivery, theranostics, and other applications. In this study, CDs were successfully prepared and isolated from PEC-GS/BG hybrids. Their chemical composition, surface functional groups, and crystal structure were comprehensively characterized. The results demonstrated that the CDs are mainly composed of carbon and oxygen. They exhibit a near-spherical morphology with an average diameter of about 7.4 nm. Then, the fluorescent properties of the CDs were thoroughly assessed. Photoluminescence (PL) measurements revealed that the CDs display intense blue fluorescence upon exposure to ultraviolet (UV) light. This emission is excitation-dependent and shows resilience to variations in pH, high ionic strength, and photobleaching. The quantum yield (QY) was determined to be around 4.5%. Additionally, the synthesized CDs exhibited excellent biocompatibility and cell-labeling capability. These findings indicate that the synthesized CDs hold significant potential for practical applications in various fields.

## 1 Introduction

Bone tissue engineering (BTE) has drawn considerable interest owing to its remarkable potential in addressing large-scale bone defects and related conditions (Peng et al., 2020; Leonovich et al., 2023). Silicate bioactive glasses (BG) are a promising material in BTE due to their outstanding biocompatibility, bioactivity, and osteoconductivity (Ke et al., 2020; Shearer et al., 2023). Zheng et al. demonstrated that BG could promote angiogenesis by stimulating the release of endogenous bioactive factors, including vascular endothelial growth factor (VEGF) (Zheng et al., 2022). The success of bone repair is highly contingent on the vascularization of the transplanted graft (Zhu et al., 2023; Pan et al., 2024). However, previous research has indicated that the osteogenic capacity of BG alone is insufficient (Putra et al., 2023).

Carbon dots (CDs) are a novel type of carbon nanomaterial, distinguished by their attractive properties that offer significant potential for a wide range of biomedical applications (Yang P. et al., 2019; Yang H. et al., 2019; Liu et al., 2019). CDs typically exhibit excitation-wavelength dependent photoluminescence emission spectra (Liu et al., 2020; Yu et al., 2022), alongside exceptional photostability and strong resistance to photobleaching (Barman et al., 2024; Zhang et al., 2023; Ghosal and Ghosh, 2019). Both *in vitro* and *in vivo* studies have demonstrated that CDs exhibit excellent cytocompatibility and biological compatibility with no apparent toxic effects (Wei et al., 2024; Dash et al., 2024; Perikala et al., 2023; Dehvari et al., 2020). Moreover, considerable investigations have been conducted into the interactions between CDs and biomacromolecules, including proteins, nucleic acids, and lipids (Li et al., 2023). Owing to these advantageous properties, CDs have been widely applied in various biomedical fields, such as bioimaging, drug delivery, and therapeutic interventions (Sun et al., 2020; Zhong et al., 2023).

Citric acid is abundant in the skeletal system, accounting for 90% of the total citric acid content in human body, and plays a crucial role in bone metabolism and formation (Książek, 2023; Wang et al., 2023). It has been shown to promote osteogenic differentiation and matrix mineralization of mesenchymal stem cells (MSCs) (Zhang et al., 2021; Wu et al., 2021). Additionally, it can also serve as a fundamental precursor for the development of CDs (Yang et al., 2022; Otten et al., 2022). In previous studies, our team incorporated citric acid into BG, resulting in the preparation of PEC-GS/BG hybrids that exhibiting enhanced bone-promoting effects (Zhao et al., 2019).

In this study, we synthesized PEC-GS/BG hybrids and identified the presence of fluorescent CDs within them. The structure and fluorescence characteristics of the CDs obtained were systematically investigated. The as-prepared CDs demonstrated near-spherical geometry, excitation-dependent emission, significant quantum yields, excellent photostability, and low toxicity. Additionally, the possible functions of the CDs in cell imaging were also explored.

## 2 Experimental section

### 2.1 Materials

Citric acid (>99%) was purchased from J&K Chemical (Beijing, China). Poly (ethylene glycol) (PEG300), methoxyethanol (99.8%), calcium 2-methoxyethoxide (CME, ≥98%) and (3-glycidoxypropyl) trimethoxysilane (GS, ≥98%) were obtained from Sigma-Aldrich (St. Louis, MO, United States). Tetraethylorthosilicate (TEOS, ≥99.0%) was acquired from Sinopharm Chemical Reagent Co., Ltd. (Shanghai, China). All commercial reagents were utilized in the form they were received, without any further purification.

### 2.2 Preparation of PEC-GS/BG hybrids and CDs

PEC-GS/BG hybrids were prepared using a previously reported procedure (Zhao et al., 2019). At a constant nitrogen flow rate, equal molar amounts of PEG300 and citric acid were introduced into a three-neck round-bottom flask. The mixture was stirred and heated to 180°C for 30 min to yield a poly (ethylene glycol-co-citric acid) (PEC) pre-polymer. The prepared PEC pre-polymers were dissolved in methoxyethanol, resulting in a 50% concentration solution. GS (molar ratio: GS/CA = 0.8/1) was added to the aforementioned solution, and the reaction was conducted at 40°C for 6 h to produce the PEC-GS pre-polymer solution.

TEOS and CME were then sequentially introduced into PEC-GS solution with an organic/inorganic mass ratio of 50:50, while ensuring a Si/Ca molar ratio of 70:30 in the inorganic phase. Specifically, TEOS and PEC-GS were first blended to achieve a colorless solution. Then, CME was added to the mixture and stirred continuously, resulting in the formation of an orange transparent solution. After 12 h of stirring, water droplets were cautiously incorporated into the mixture until a significant increase in viscosity was observed. The mixture was subsequently transferred to a Teflon mold and allowed to gel at room temperature for 24 h, then dried in an oven at 60°C for 7 days. The resulting product is referred to as PEC-GS/BG hybrids.

Subsequently, the hybrids were immersed in distilled water, and CDs were gradually released as the material degraded. The degraded solution, containing CDs, was filtered twice using a 0.22 μm membrane to eliminate any remaining bulk particles. The filtered solution was ultimately dried at 45°C to obtain the CDs.

### 2.3 Apparatus

Fourier transform infrared (FTIR) spectroscopic measurements were performed using a Bruker VECTOR22 spectrometer (Bruker, Karlsruhe, Germany). X-ray photoelectron spectroscopy (XPS) for elemental analysis was conducted with equipment from Thermoelectricity Instruments, United States. Corresponding energy dispersive X-ray spectroscopy (EDS) analysis was carried out using an EDS detector integrated with a Hitachi S-4800 high-resolution field emission scanning electron microscope. Transmission electron microscope (TEM) images were obtained with an FEI Tecnai G20 (FEI, Hillsboro, Oregon, United States) operating at an acceleration voltage of 200 kV. Dynamic light scattering (DLS) experiments were performed in water at room temperature using a 90 Plus particle size analyzer from Brookhaven Instruments Corp (Holtsville, NY, United States). Ultraviolet-visible (UV-vis) absorption spectra were recorded using a UV-2450 UV-vis spectrophotometer (Shimadzu, Tokyo, Japan). Photoluminescence (PL) spectra were measured at room temperature with an FL-7000 spectrophotometer (Hitachi, Tokyo, Japan). Cell imaging was conducted with an Inverted-BX51 microscope (Olympus, Melville, NY, United States) that utilized a 488 nm laser.

### 2.4 Assessment of quantum yields

The quantum yield (QY) of the CDs was determined with quinine sulfate (dissolved in 0.1 M H_2_SO_4_, QY = 54%) serving as a reference. The QY was calculated according to the following equation (Shen et al., 2021):

$$\varphi =\varphi \prime \frac{A^{\prime }}{I^{\prime }}\frac{I}{A}{\left(\frac{n}{n^{\prime }}\right)}^{2}$$

In this equation, *φ* and *φ′*, *I* and *I′*, *A* and *A′* represent the QY, fluorescence intensity, and absorbance of the obtained CDs and the quinine sulfate solution, respectively. Both *n* and *n’* represent the refractive indices of water. Modify the concentrations of the quinine sulfate and CDs solutions to ensure their optical absorbance was below 0.05.

### 2.5 Cellular toxicity test

The cytotoxicity of CDs was evaluated using an MTT assay (Yang et al., 2023; Zhang W. et al., 2024) on MC3T3-E1 cells, which were obtained from the Cell Bank of the Chinese Academy of Sciences. Initially, MC3T3-E1 cells (100 μL) were seeded into 96-well culture plates at a density of 1.0 × 10^5^ cells/mL in α-MEM complete medium. After 12 h, the medium was replaced with fresh medium containing various concentrations of CDs (0, 10, 20, 50, 100, 200, and 400 *μ*g/mL), maintaining a total volume of 200 *μ*L per well. Following another 24 h of incubation, 10 *μ*L of MTT solution (5 mg/mL) was added to each well and incubated for another 4 h. Subsequently, the culture medium was discarded, and 100 *μ*L of DMSO was introduced to dissolve the formazan crystals. The absorbance of each well was measured at a specific wavelength using a microplate reader. Cytotoxicity was assessed using the formula below:

$$\text{Cell viability }\left(\%\right)=A_{1}/A_{2}\times 100$$

where A_1_ is the absorbance of the wells containing cells exposed to the CDs, and A_2_ is the absorbance of the wells containing cells not exposed to the CDs. Each control and test concentration was assessed in six replicate wells. Results are presented as means with standard deviations.

### 2.6 Cell imaging

The potential of CDs for biolabeling was assessed through cell imaging of MC3T3-E1 cells (Sobhanan et al., 2023). In brief, MC3T3-E1 cells were cultured in complete α-MEM medium containing 10% fetal bovine serum, 100 *µ*g/mL penicillin, and 100 *µ*g/mL streptomycin. The culture was maintained at 37°C in a 5% CO_2_ atmosphere. Cells were seeded into well plates at a concentration of 5.0 × 10^5^ cells/mL and incubated for 24 h. Subsequently, cells were incubated with 100 *μ*g/mL CDs for 2 h and washed three times with phosphate-buffered saline (PBS). Fluorescence imaging was then performed using an Inverted-BX51 confocal fluorescence microscope with a ×20 objective lens and a 488 nm excitation laser.

## 3 Results and discussion

### 3.1 Structural characterization

**FIGURE 1:**
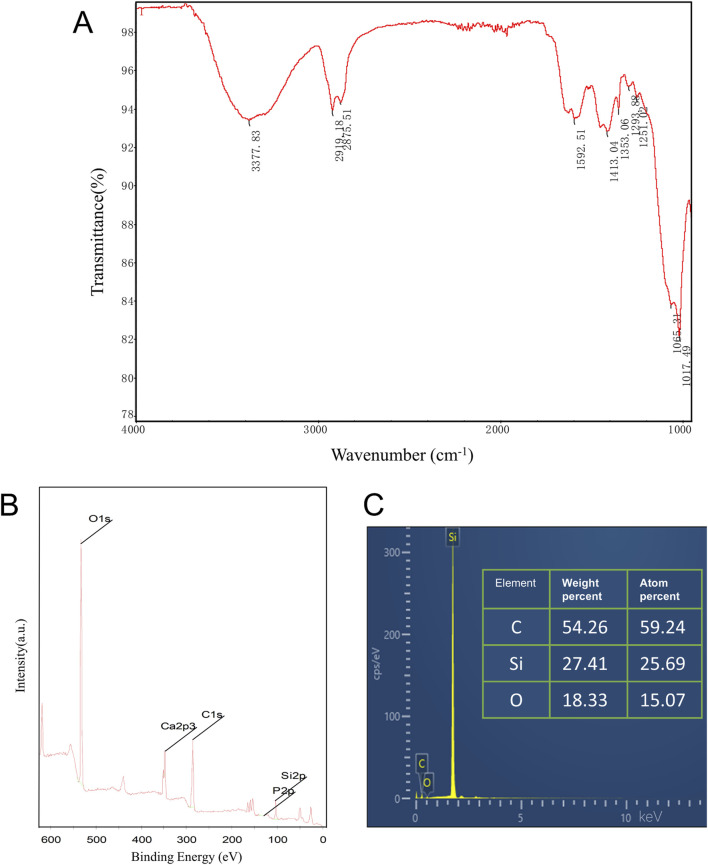
**(A)** Fourier transform infrared (FTIR) spectrum, **(B)** X-ray photoelectron spectroscopy (XPS) survey scan and **(C)** energy dispersive X-ray spectroscopy (EDS) of CDs.

The functional groups of CDs were characterized by FT-IR spectrum (Figure 1A). The broad peak around 3,400 cm^−1^ is attributed to the N-H or O-H stretching vibrations in amino and hydroxyl groups. The peaks at 2,919 cm^−1^ and 2,875 cm^−1^ correspond to the stretching vibrations of the C-H bond. The peak at 1,592 cm^−1^ can be assigned to the stretching vibration of the C=O bond, while the peaks at 1,413 cm^−1^ and 1,353 cm^−1^ are due to the stretching vibrations of the C-N bond. The peaks at 1,065 cm^−1^ and 1,017 cm^−1^ are attributed to the C-O bond. The surface composition and elemental analysis of the obtained CDs were characterized using XPS and EDS. The XPS results are shown in Figure 1B. A survey scan was conducted from 0 to 600 eV, revealing primary peaks at 100, 285, 350, and 532 eV, corresponding to Si2p, C1s, Ca2p, and O1s, respectively (with hydrogen not detectable by XPS). Additionally, the EDS results (Figure 1C) indicate that the CDs primarily consist of carbon and oxygen (with a C/O weight ratio of 2.96), along with detectable amounts of silicon. These findings are consistent with the XPS results and suggest the presence of numerous hydrophilic groups on the CD surfaces. The hydrophilic groups evidently stabilize the CDs in aqueous solutions.

**FIGURE 2:**
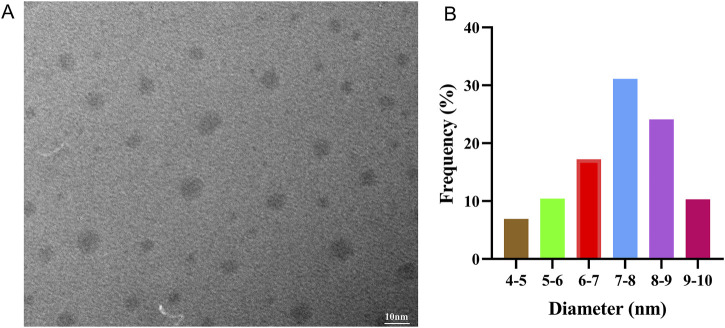
**(A)** Typical TEM image and **(B)** corresponding particle size distribution of as prepared CDs.

The size of nanoparticles is crucial for bio-applications (Hu et al., 2022). The TEM image (Figure 2A) demonstrates that the CDs are nearly spherical and well-dispersed. Statistical analysis of the grain size based on the TEM images indicates that the CDs have a size range of 4–10nm, with an average diameter of 7.4 ± 1.4 nm (Figure 2B). However, DLS measurements indicated that the hydrodynamic diameter of the CDs is approximately 24 nm, which is larger than the size determined by TEM. This discrepancy is attributed to the impact of hydration in a water-based solution. While TEM measures the diameter of CDs after being dried on a surface, DLS determines the diameter of hydrated CDs in the solution, reflecting their more “swollen” state (Hu et al., 2022). Therefore, the size of the obtained CDs is appropriate for biological applications. Moreover, the zeta potential of the CDs was measured to be −15.3 mV.

### 3.2 Fluorescent properties

**FIGURE 3:**
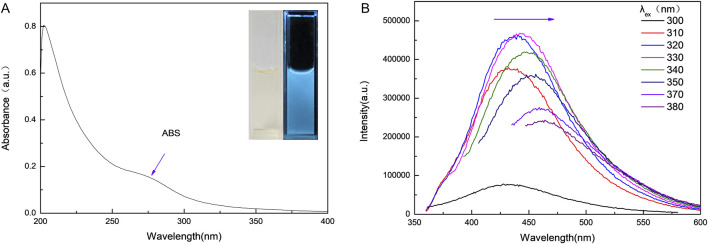
**(A)** UV-vis absorption spectrum of CDs. Inset: photographs of the CD aqueous solutions under visible light (left) and UV light (right), respectively; **(B)** Excitation dependent photoluminescence behavior of CDs.

We measured the UV-vis absorption spectrum of the CDs to determine their band structure. The absorption spectrum, shown in Figure 3A, features two peaks in the 200–300 nm region. The peak at 275 nm is likely due to the π-π* transition of the CDs, while the peak at 207 nm may result from the generation of multiple polyaromatic chromophores. Additionally, as depicted in Figure 3B, the emission spectrum of the CDs was recorded upon excitation with light in the range of 300–380 nm. Both the emission intensity and wavelength are dependent on the excitation wavelength. The emission intensity increases gradually with the excitation wavelength, peaking at 330 nm, and then declines as the excitation wavelength continues to increase. Consistent with previous studies, the emission of the CDs exhibits a notable red-shift property, where the emission wavelength shifts to longer wavelengths as the excitation wavelength increases. This excitation-dependent emission is an inherent characteristic of CDs and has been extensively documented in the literature (Zhang Y. et al., 2024). In addition, the QY of the CDs was calculated to be approximately 4.5% using quinine sulfate solution as a reference.

The PL origin of CDs remains under debate, with potential explanations including surface defect states, quantum confinement effects, or other factors. The notion that functional groups act as continuous surface defect states and govern emission properties has gained broad acceptance in the scientific community (Dorontic et al., 2022). The surface defect states on CDs are varied, resulting in heterogeneous emission energy levels and producing excitation-tunable emission (Kaur et al., 2024). Ding et al. also suggested that the photoluminescence of CDs is likely dominated by continuous surface defect states, with different defect states contributing to emissions at different wavelengths (Ding et al., 2024). However, Zhang et al. concluded in their research that the functional groups are non-radiative surface states (Zhang P. et al., 2024). The quantum confinement effect, which is also referred to as the size effect, stands as another broadly recognized mechanism model (Vargas-Reyes et al., 2024). Previous studies have suggested that the strong PL of CDs, observed upon surface passivation, is due to the quantum confinement effect of emissive energy traps on the CD surface (Ayisha Naziba et al., 2024). Rao et al. proposed that the excitation-tunable PL emission of CDs is mainly due to variations in size, rather than the presence of different emission trap sites on particles of similar size (Rao et al., 2023). However, research has indicated that CDs of varying sizes exhibited identical PL emission peaks under a 365 nm UV lamp, which is unexpected given the typical size-dependent emission behavior (Gan et al., 2016). In this study, the excitation wavelength dependence of CD photoluminescence may be attributed to variations in particle size. However, the exact mechanism behind the PL of CDs remains controversial and requires further investigation.

**FIGURE 4:**
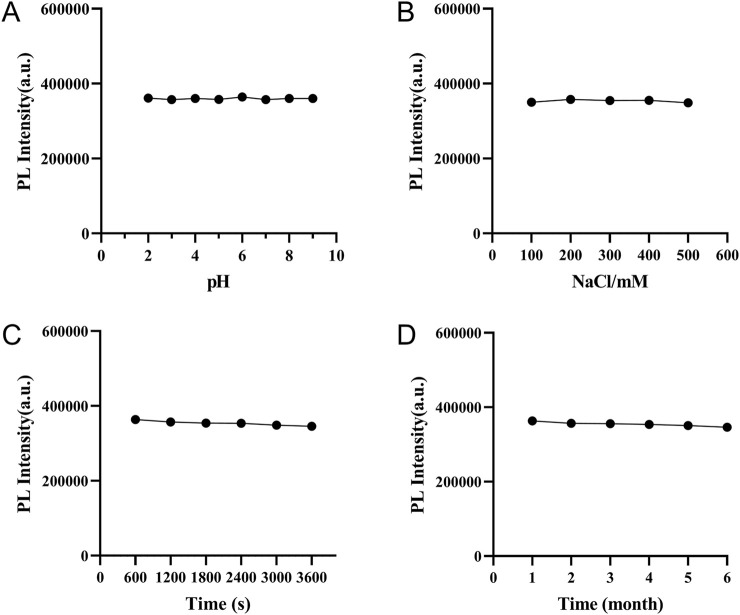
**(A)** Effect of pH on the photoluminescence intensity of CDs; **(B)** Fluorescence intensity of CDs in NaCl aqueous solution (pH = 7) against the ionic strength; **(C)** Dependence of the fluorescence intensity of CDs on excitation time under 488 nm irradiation in ultrapure water; **(D)** Effect of storage time on the photoluminescence intensity of CDs.

The stability of the as-prepared CDs under various conditions was thoroughly examined. The PL intensity of the CDs exhibited pH independence over a broad range of 2–9, as shown in Figure 4A. Furthermore, the PL intensity remained nearly constant in solutions with NaCl concentrations up to 500 mM, as depicted in Figure 4B. The CDs also demonstrated remarkable stability under UV excitation, with no significant change in PL intensity after 1 hour (Figure 4C), and maintained their PL intensity after 6 months of storage (Figure 4D). These findings indicate that the CDs possess exceptional stability, highlighting their potential for biological applications.

### 3.3 Cytotoxicity and cell-imaging

**FIGURE 5:**
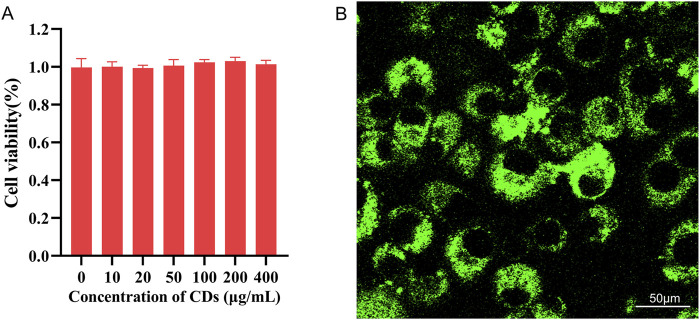
**(A)** Cell viability of MC3T3-E1 cells after 24-h incubation with various concentrations of CDs; **(B)** Fluorescence images of MC3T3-E1 cells incubated with CDs for 2 h under 488 nm filter irradiation. Scale bar:50 *μ*m.

Cytotoxicity is a critical parameter for evaluating the biocompatibility of biomaterials in cell experiments. In this study, standard MTT assays were performed on MC3T3-E1 cells to assess the cytotoxicity of the synthesized CDs (Yang et al., 2023; Zhang W. et al., 2024). Figure 5A illustrates cell viability following 24-h incubation with CDs at concentrations of 10, 20, 50, 100, 200, and 400 *μ*g/mL. The results clearly indicate that the CDs exhibit low cytotoxicity, even at concentrations as high as 200 *μ*g/mL. Therefore, these CDs demonstrate significant potential for single-molecule imaging and tracking within living cells. To further investigate this application, confocal fluorescence imaging was employed. As shown in Figure 5B, the green emission is predominantly localized within the cytoplasmic region, indicating that the CDs successfully penetrate the cell membrane. The uniform and regular distribution of CDs within the cellular matrix suggests an enhancement in cellular biological activity. These initial findings indicate that the synthesized CDs are promising candidates for use in cell imaging, drug delivery, and bone tissue engineering.

## 4 Conclusion

In the present study, carbon dots (CDs) were successfully synthesized and isolated from PEC-GS/BG hybrids. The CDs exhibit near-spherical geometry with an average diameter of approximately 7.4 nm. They demonstrate strong blue luminescence under ultraviolet irradiation. As the excitation wavelength increases, the emission intensity initially increases and then gradually decreases, with a concurrent red shift in the emission wavelength. Moreover, the CDs exhibit excellent stability and a quantum yield of approximately 4.5%. Cytotoxicity and cell imaging experiments indicate that the CDs exhibit high biocompatibility and uniform intracellular distribution, making them promising candidates for cell-labeling applications.

## Data Availability

The original contributions presented in the study are included in the article/supplementary material, further inquiries can be directed to the corresponding authors.
